# Infusion of etoposide in the CA1 disrupts hippocampal immediate early gene expression and hippocampus-dependent learning

**DOI:** 10.1038/s41598-022-17052-y

**Published:** 2022-07-27

**Authors:** Sydney Weber Boutros, Kat Kessler, Vivek K. Unni, Jacob Raber

**Affiliations:** 1 Department of Behavioral Neuroscience, OHSU, 3181 SW Sam Jackson Park Rd, Portland, OR 97239 USA; 2 Department of Neurology, OHSU, 3181 SW Sam Jackson Park Rd, Portland, OR 97239 USA; 3 Jungers Center for Neurosciences Research, OHSU Parkinson Center, OHSU, 3181 SW Sam Jackson Park Rd, Portland, OR 97239 USA; 4 Departments of Psychiatry and Radiation Medicine, OHSU, 3181 SW Sam Jackson Park Rd, Portland, OR 97239 USA; 5 Division of Neuroscience, ONPRC, 505 NW 185th Ave, Beaverton, OR 97006 USA

**Keywords:** Neuroscience, Medical research

## Abstract

Tight regulation of immediate early gene (IEG) expression is important for synaptic plasticity, learning, and memory. Recent work has suggested that DNA double strand breaks (DSBs) may have an adaptive role in post-mitotic cells to induce IEG expression. Physiological activity in cultured neurons as well as behavioral training leads to increased DSBs and subsequent IEG expression. Additionally, infusion of etoposide—a common cancer treatment that induces DSBs—impairs trace fear memory. Here, we assessed the effects of hippocampal infusion of 60 ng of etoposide on IEG expression, learning, and memory in 3–4 month-old C57Bl/6J mice. Etoposide altered expression of the immediate early genes *cFos* and *Arc* in the hippocampus and impaired hippocampus-dependent contextual fear memory. These data add to the growing evidence that DSBs play an important role in IEG expression, learning, and memory, opening avenues for developing novel treatment strategies for memory-related disorders.

## Introduction

Immediate early genes (IEGs)—including the proto-oncogene c*Fos* and activity-regulated cytoskeleton-associated protein (*Arc)*—are important for synaptic plasticity and learning and memory^1–3^. At baseline, these genes are expressed at low basal levels. Activity within neurons leads to a rapid increase in their expression and a subsequent return to baseline levels within hours^4^. This precise timing is important for accurate encoding and recall, especially related to hippocampus-dependent contextual learning and memory^5–7^. Yet, what exactly leads to the rapid expression of these genes is still unclear.

Recently, evidence has emerged that DNA double strand breaks (DSBs) may contribute to IEG expression^8,9^. DSBs are observed after physiological neuronal activation^8^, and are associated with an increase in mRNA levels of a select sub-set of genes, many of which are part of the IEG family^9^. Contextual fear conditioning also induces DSBs in neuronal and non-neuronal cells that leads to transcriptional changes in neurons and glia^10^. Notably, the timing of DSB induction and their proper repair are important components of this process. Mice carrying mutations that cause Alzheimer’s disease show higher basal levels of DSBs and impaired DSB repair compared to wild-type mice, measured by a common DSB repair marker, γH2Ax^8^. Post-mortem analysis in people reflects this as well: patients with Alzheimer’s disease have higher γH2Ax than those with mild cognitive impairment or cognitively unimpaired age-matched controls^11^.

In attempts to identify causal links, the chemotherapy agent etoposide, which induces DSBs by interfering with the topoisomerase-II beta complex, is being used^12^. We have recently shown that systemic administration of etoposide impairs long-term contextual and cued fear memory and changes hippocampal FosB protein levels^13^. In the clinic, etoposide’s effects on learning and memory are confounded by the presence of many other variables but do generally point towards impaired hippocampus-dependent cognitive performance^14^.

Yet, etoposide’s penetration past the blood brain barrier is poor (< 3%)^15^, and toxicity is seen in cell cultures at doses around 10 µg/mL^16^. As such, direct infusions into the central nervous system are used in patients being treated for brain tumors. Cerebral spinal fluid levels were around 3 µg/mL in patients 2 h after receiving 0.5 mg intraventricular infusions^17^; while headaches were the main side effects reported, there was no assessment of learning or memory. In mice, direct infusion of 0.1 µg/µL (but not 1 ng/µL) of etoposide in the prelimbic area impairs long-term fear memory and alters the timeline of *cFos*, *Arc*, *Npas4*, and *Cyr61* RNA expression^18^. More research is needed to identify the extent and specificity of DSBs in learning and memory as well as characterize the molecular changes induced by lower doses. Namely, testing the effects of etoposide in brain regions known to be important for learning and memory—such as the hippocampus—is an important next-step in understanding the role of DSBs in the learning and memory process, as well as testing lower doses similar to those observed in the clinic. Further clarifying possible mechanisms, like disruption to IEG expression, is also needed for a better understanding of this system.

Here, we used 60 ng of etoposide to induce DSBs selectively in the CA1 region of the hippocampus during fear acquisition. We hypothesized that CA1 infusions of etoposide would interfere with hippocampal IEG expression and impair hippocampus-dependent long-term contextual (but not hippocampus-independent cued) fear memory. Etoposide increased γH2Ax in the CA1, but not in other regions of the hippocampus. Etoposide also impaired *cFos* expression throughout the hippocampus and decreased long-term contextual-dependent freezing. These data point to a nuanced disruption of intra-hippocampal signaling by etoposide and provide more direct causal evidence that DSBs are involved in hippocampus-dependent learning and memory via regulation of IEGs.

## Results

### Fear conditioning and hippocampal injection of etoposide increases γH2Ax foci in the CA1

Using male C57Bl/6J (WT) mice, we bilaterally implanted cannula targeting the CA1 region of the hippocampus. Body weights were used to track health and recovery of mice throughout all experiments (Table 1). To determine the effects of etoposide on hippocampal IEGs and hippocampus-dependent learning and memory, we bilaterally infused 0.3 µL of etoposide (0.1 µg/µL) or saline into the CA1 region of the hippocampus in male WT mice. Two hours later, mice were euthanized (behaviorally naïve, *n* = 4–5/treatment) or trained in a fear conditioning paradigm and immediately euthanized (*n* = 4/treatment).

**Table 1:** Body weights before cannula implantation (“Pre-Surgery”) and at the end of the study (“Post-Infusion”) in male and female mice.

Sex	Time and treatment			
	Pre-surgery		Post-infusion	
	Saline	Etoposide	Saline	Etoposide
Male	24.33 ± 0.39	24.13 ± 0.38	28.00 ± 0.43	26.35 ± 0.63
Female	19.28 ± 0.46	18.86 ± 0.46	19.88 ± 0.60	20.35 ± 0.29

Data presented as averages ± SEMs.

**Figure 1:**
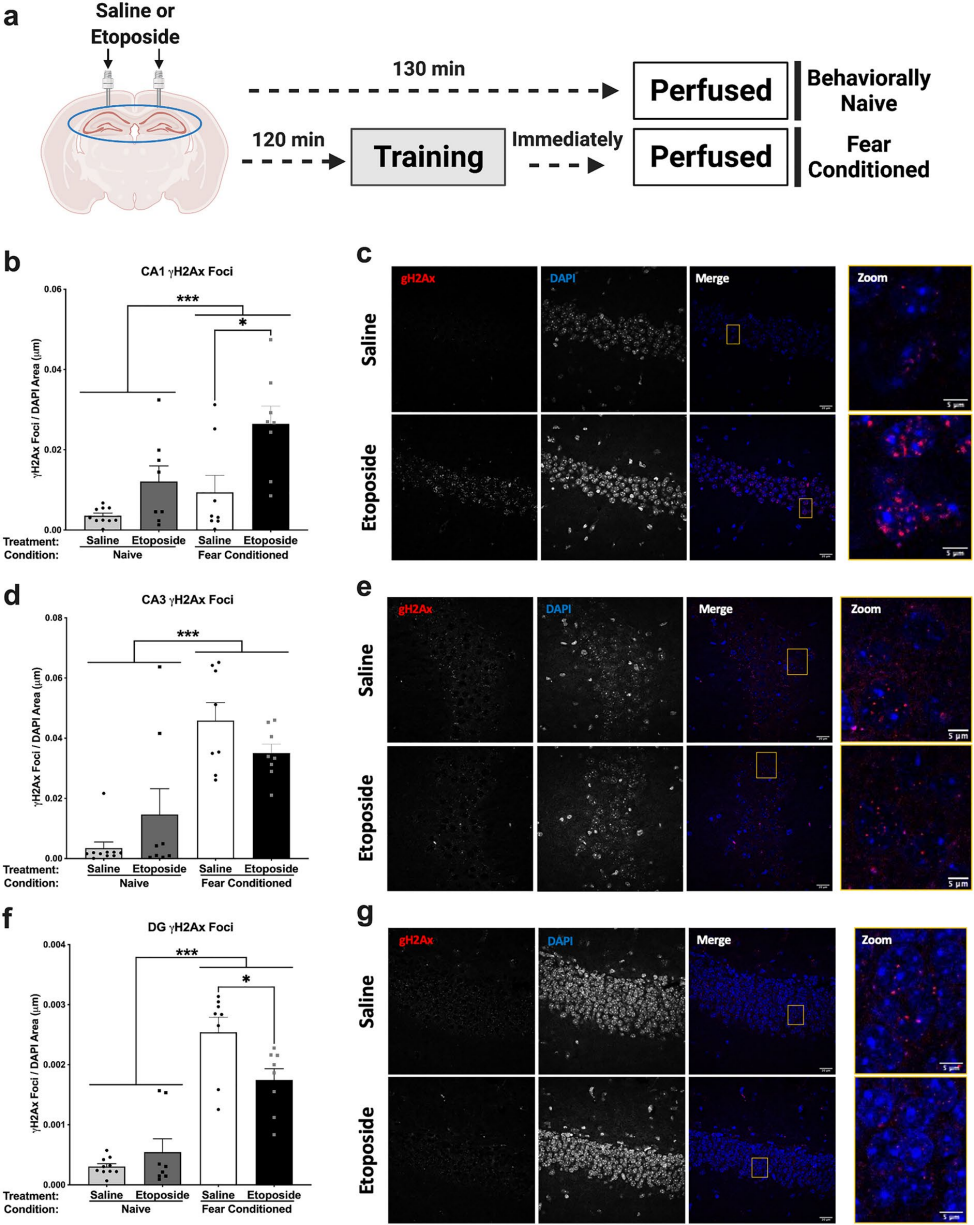
Analysis of γH2Ax in the hippocampus following infusions of saline or etoposide and fear conditioning. (**a**) Schematic of the experimental timeline (*n* = 4–5/behavioral group/treatment). (**b**) γH2Ax foci in CA1. Fear conditioning significantly increased the number of γH2Ax foci (*F*(1,30) = 12.599, *p* = 0.001). Etoposide infusions significantly increased the number of γH2Ax foci (*F*(1,30) = 20.587, *p* < 0.001). (**c**) Representative images of γH2Ax staining in the CA1 from fear conditioned mice. The far-right panel is zoomed in on the yellow box in the “merge” image. (**d**) γH2Ax foci in CA3. Fear conditioning significantly increased γH2Ax foci (*F*(1,30) = 53.260, *p* < 0.001). Etoposide infusions did not change the number of γH2Ax foci (*F*(1,30) = 0.003, *p* = 0.995). There was a significant interaction between behavior and treatment groups (*F*(1,30) = 4.689, *p* = 0.038). (**e**) Representative images of γH2Ax staining in the CA3. (**f**) γH2Ax foci in the DG. Fear conditioning significantly increased γH2Ax foci (*F*(1,30) = 128.782, *p* < 0.001). There was a significant interaction between behavior condition and treatment (*F*(1,30) = 9.203, *p* = 0.005), where etoposide infusions significantly decreased the number of γH2Ax foci in the fear conditioned group (*p* < 0.05) but increased it in the naïve group. (**g**) Representative images of γH2Ax staining in the DG. Data are presented as total number of foci normalized to area of DAPI (mm) ± SEM. *p < 0.05.

We first assessed the effects of etoposide infusions on γH2Ax formation by immunofluorescence in the CA1, CA3, and dentate gyrus (DG) regions of the hippocampus (Fig. 1a). Mice trained in fear conditioning had significantly more γH2Ax foci in the CA1 than behaviorally naïve animals (*p* = 0.001; Fig. 1b). Additionally, etoposide infusions increased the number of γH2Ax foci in the CA1 region (*p* < 0.001; Fig. 1b). Sidak’s post hoc testing indicated that mice that received fear conditioning and etoposide infusions had significantly more γH2Ax than fear conditioned, saline-infused mice (*p* < 0.05). Representative images of γH2Ax in the CA1 can be seen in Fig. 1c.

Similarly, γH2Ax foci in the CA3 were significantly increased in animals that underwent fear conditioning (*p* < 0.001; Fig. 1d). There was no difference between saline- and etoposide-infused animals in the CA3 region (*p* = 0.995), though we found a significant condition by treatment interaction (*p* = 0.038; Fig. 1d), where etoposide increased γH2Ax in the behaviorally naïve mice but decreased it in the fear conditioned mice. Representative images of γH2Ax in the CA3 can be seen in Fig. 1e.

Analysis of γH2Ax foci in the DG also revealed a significant effect of behavioral condition (*p* < 0.001; Fig. 1f), as well as a significant condition by treatment interaction (*p* = 0.005). Sidak’s post hoc testing indicated that fear conditioned, etoposide-infused mice had less γH2Ax than conditioned-matched, saline-infused mice (*p* < 0.05). There was no main effect of treatment (*p* = 0.140). Representative images of γH2Ax in the DG can be seen in Fig. 1g. These region- and condition-dependent change in γH2Ax focus formation suggests that CA1-targeted infusion of etoposide disrupt DSBs in the hippocampus at baseline and during a learning event.

To confirm that our infusions were not causing cell death, we assessed Fluoro-Jade C staining in the hippocampus. There was no visible sign of Fluoro-Jade C staining in any hippocampal regions, indicating that neither saline nor etoposide infusions caused cell death (Supplemental Fig. 1a,b).

### Etoposide disrupts timeline of Arc and cFos expression in the hippocampus

**Figure 2:**
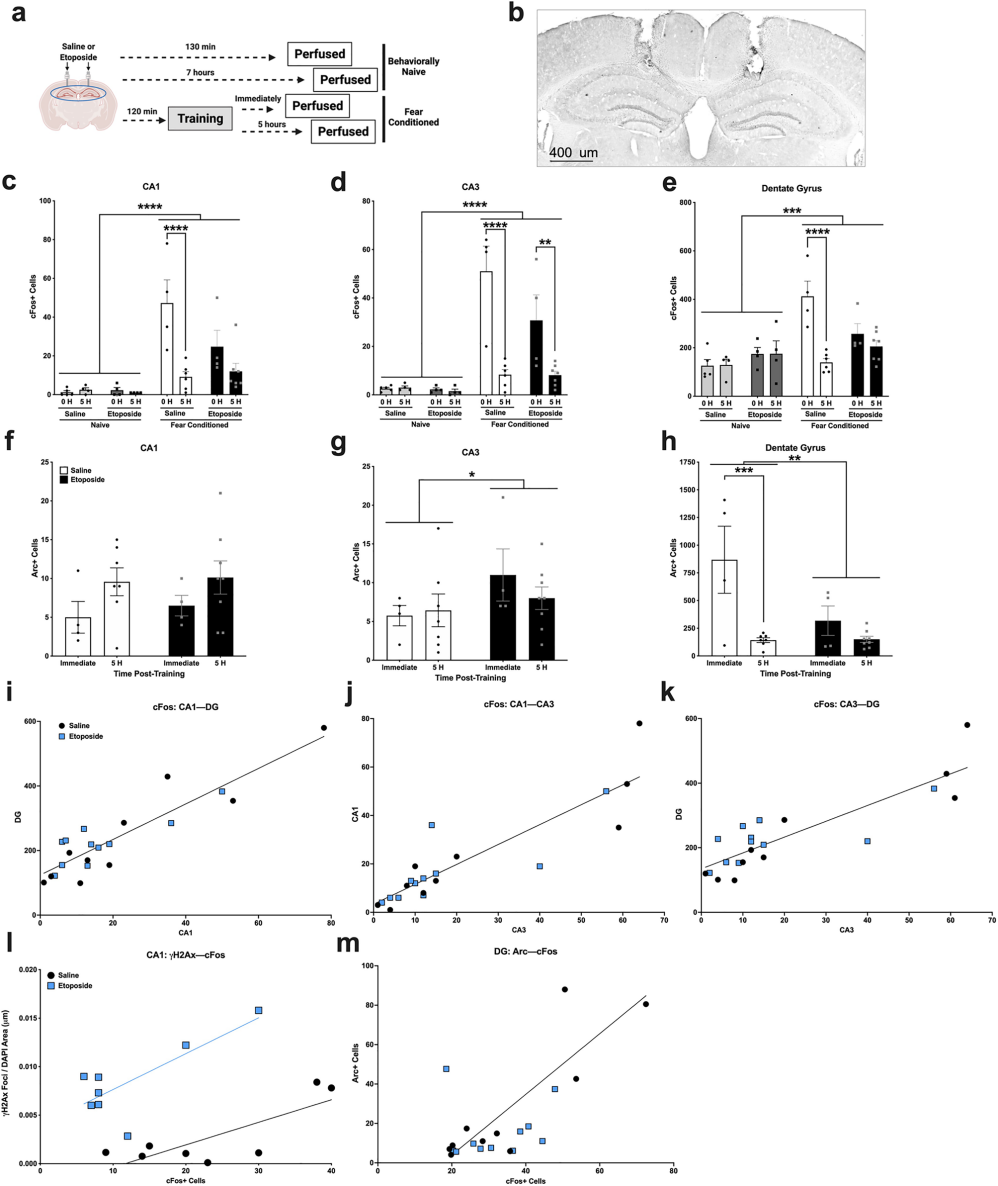
Analysis of cFos and Arc by immunohistochemistry in the hippocampus at two time points after fear conditioning. (**a**) Schematic of the experimental timeline (*n* = 4–7/behavior group/treatment/time point). (**b**) Representative overview image to show cannula placement. (**c**) Total number of cFos + cells in the CA1. There were more cFos + cells in animals that underwent fear conditioning (*F*(1,30) = 43.165, *p* < 0.001), and in the group euthanized immediately post-training (*F*(1,30) = 12.608, *p* = 0.001). There also was a significant time post-training by behavior interaction (*F*(1,30) = 13.769, *p* < 0.001). Sidak’s post-hoc comparison indicated that the fear conditioned saline group euthanized 5 h post-training had significantly fewer cFos + cells compared to the immediately euthanized group (*p* < 0.0001), but the 5 h post-training etoposide group was not different than the immediate group (*p* = 0.2607). (**d**) Total number of cFos + cells in the CA3. There were more cFos + cells in the animals that underwent fear conditioning (*F*(1,30) = 43.071, *p* < 0.001), and in the the group euthanized immediately post-training (*F*(1,30) = 23.265, *p* < 0.001). There also was a significant time post-training by behavior interaction (*F*(1,30) = 23.747, *p* < 0.001). Sidak’s post hoc comparison indicated that the fear conditioned saline group euthanized 5 h post-training had significantly fewer cFos + cells compared to the immediately euthanized group (*p* < 0.0001), and that the fear conditioning etoposide group euthanized 5 h post-training had fewer cFos + cells compared to the immediately euthanized group (*p* = 0.0037). (**e**) Total number of cFos + cells in the DG. There were more cFos + cells in the animals that underwent fear conditioning (*F*(1,30) = 22.965, *p* < 0.001). There was also a significant time by treatment interaction (*F*(1,30) = 4.583, *p* = 0.041), time by behavior interaction (*F*(1,30) = 4.940, *p* = 0.034), treatment by time by behavior interaction (*F*(1,30) = 7.441, *p* = 0.011), and a trend towards a time by treatment interaction (*F*(1,30) = 4.583, *p* = 0.052). Sidak’s post hoc testing revealed a significant decrease in the fear conditioned saline-infused group 5 h post-training compared to immediately post-training (*p* < 0.0001), but not in the fear conditioned etoposide-infused groups (*p* = 0.7132). (**f**) Total number of Arc + cells in the CA1. There was a significant increase in Arc + cells in animals euthanized 5 h post-training compared to immediately post-training (*F*(1,42) = 5.054, *p* = 0.030). (**g**) Total number of Arc + cells in the CA3. Mice infused with etoposide had overall more Arc + cells (*F*(1,42) = 5.067, *p* = 0.023). (**h**) Total number of Arc + cells in the DG. There was a significant effect of time post-training (*F*(1,42) = 22.104, *p* < 0.001), treatment (*F*(1,42) = 8.362, *p* = 0.006), and a time by treatment interaction (*F*(1,42) = 8.222, *p* = 0.006. Sidak’s post hoc test showed that the number of Arc + cells dropped over time in the saline-infused groups (*p* < 0.001), but not the etoposide-infused groups (*p* = 0.539).(**i**) A significant positive correlation between the total number of cFos + cells in the CA1 and DG after fear conditioning (*F*(1,19) = 83.13, *p* < 0.0001). There was no difference between the saline- and etoposide-infused mice. (**j**) A significant positive correlation between the total number of cFos + cells in the CA1 and CA3 (*F*(1,19) = 67.62, *p* < 0.0001). There was no difference between the saline- and etoposide-infused mice. (**k**) A significant positive correlation between the total number of cFos + cells in the CA3 and DG (*F*(1,19) = 56.16, *p* < 0.0001). There was no difference between the saline- and etoposide-infused mice. (**l**) Correlation between average number of γH2Ax normalized to DAPI area in the CA1 and the total number of cFos + cells in the CA1. There was a significant positive correlation in both the fear conditioned saline-infused animals (*F*(1,6) = 10.53, *p* = 0.0176) and the fear conditioned etoposide-infused animals (*F*(1,6) = 9.012, *p* = 0.0239). (**m**) Correlation between cFos and Arc cells in the DG. There was a significant positive correlation in the fear conditioned saline-infused animals (*F*(1,8) = 24.23, *p* = 0.0012), but not in the fear conditioned etoposide-infused animals *(F*(1.8) = 0.089, *p* = 0.7724)*.* Data are presented as total number of immunopositive cells ± SEM. *p < 0.05, **p < 0.01, ***p < 0.001, ****p < 0.0001.

Induction of DSBs with etoposide in the prelimbic area of mice interfere with the timeline of IEG expression, increasing expression 5 h after stimulation^18^. Thus, we looked at two IEGs—*Arc* and *cFos*—using immunohistochemistry immediately following fear training (2 h after infusion, *n* = 4/treatment) or 5 h after training (7 h after infusion, *n* = 7–8/treatment; Fig. 2a) in male WT mice. Additionally, we analzyed *cFos* in behaviorally naïve animals that received infusions of saline or etoposide (*n* = 4–5/treatment). A representative image of the cannula location and hippocampal regions is illustrated in Fig. 2b. Representative images of cFos and Arc staining in the CA1, CA3, and DG can be seen in Supplementary Fig. 2a–f.

In the CA1, mice that underwent fear conditioning had significantly more cFos + cells than behaviorally naïve mice (*p* < 0.001), and mice euthanized immediately after fear conditioning had more than mice euthanized 5 h later (*p* = 0.001; Fig. 2c). We also found a significant time post-training by behavior interaction (*p* < 0.001). Sidak’s post-hoc comparison indicated that fear conditioned, saline-infused mice euthanized 5 h post-training had significantly fewer cFos + cells compared to the immediately-euthanized, fear conditioned saline group (*p* < 0.0001). However, this was blunted in the fear-conditioned etoposide-infused groups: mice euthanized 5 h post-training were not different than mice euthanized immediately (*p* = 0.2607; Fig. 2c).

Similarly, fear conditioning significantly increased the amount of cFos in the CA3 (*p* < 0.001; Fig. 2d). We also found a time post-training by behavior interaction in the CA3 (*p* < 0.001). Sidak’s post-hoc comparison showed that both saline- and etoposide-infused animals euthanized immediately after fear conditioning had more cFos + cells than mice euthanized 5 h after fear conditioning (*p* < 0.0001, *p* = 0.0037, respectively; Fig. 2d).

There were also more cFos + cells in the DG in fear conditioned animals than behavioral naïve animals (*p* < 0.001; Fig. 2e). We found significant time by treatment (*p* = 0.041), time by behavior condition (*p* = 0.034), and treatment by time by behavior condition (*p* = 0.011) interactions, as well as a trend towards a time by treatment interaction (*p* = 0.052). Sidak’s post hoc test indicated that only the fear conditioned, saline-infused groups showed a drop in the number of cFos + cells (*p* < 0.0001); this pattern was absent in the fear conditioned, etoposide-infused groups (*p* = 0.7132; Fig. 2e).

We next analyzed the number of Arc + cells in the CA1, CA3, and DG in mice that underwent fear conditioning with saline or etoposide infusions. In the CA1, we found a significant effect of time (*p* = 0.030), though in the opposite direction compared to the number of cFos + cells (Fig. 2f). Both saline- and etoposide-infused mice euthanized 5 h after training had more Arc + cells than the immediate groups. In the CA3, the number of Arc + cells was affected by treatment (*p* = 0.023), but not time (*p* = 0.414), with the etoposide-infused groups showing more Arc + cells than the saline-infused groups (Fig. 2g). Lastly, the number of Arc + cells in the DG was affected by time (*p* < 0.001), treatment (*p* = 0.006), and a time x treatment interaction (*p* = 0.006; Fig. 2h). Sidak’s post-hoc testing indicated that the number of Arc + cells dropped over time in the saline-infused groups (*p* < 0.001) but not the etoposide-infused groups (*p* = 0.539). Overall, the etoposide groups had fewer Arc + cells (Fig. 2h).

To assess if communication between hippocampal regions was disrupted by etoposide, we ran correlations of the number of cFos + cells between the CA1, CA3, and DG. We found significant positive correlations for both saline- and etoposide-treated animals between the CA1 and DG (*p* < 0.0001; Fig. 2I), the CA1 and CA3 (*p* < 0.0001; Fig. 2j), and the CA3 and DG (*p* < 0.0001; Fig. 2k). Similar analysis of intra-hippocampal Arc signal did not reveal any significant correlations between regions in either treatment group (Supplemental Fig. 3a–c).

**Figure 3:**
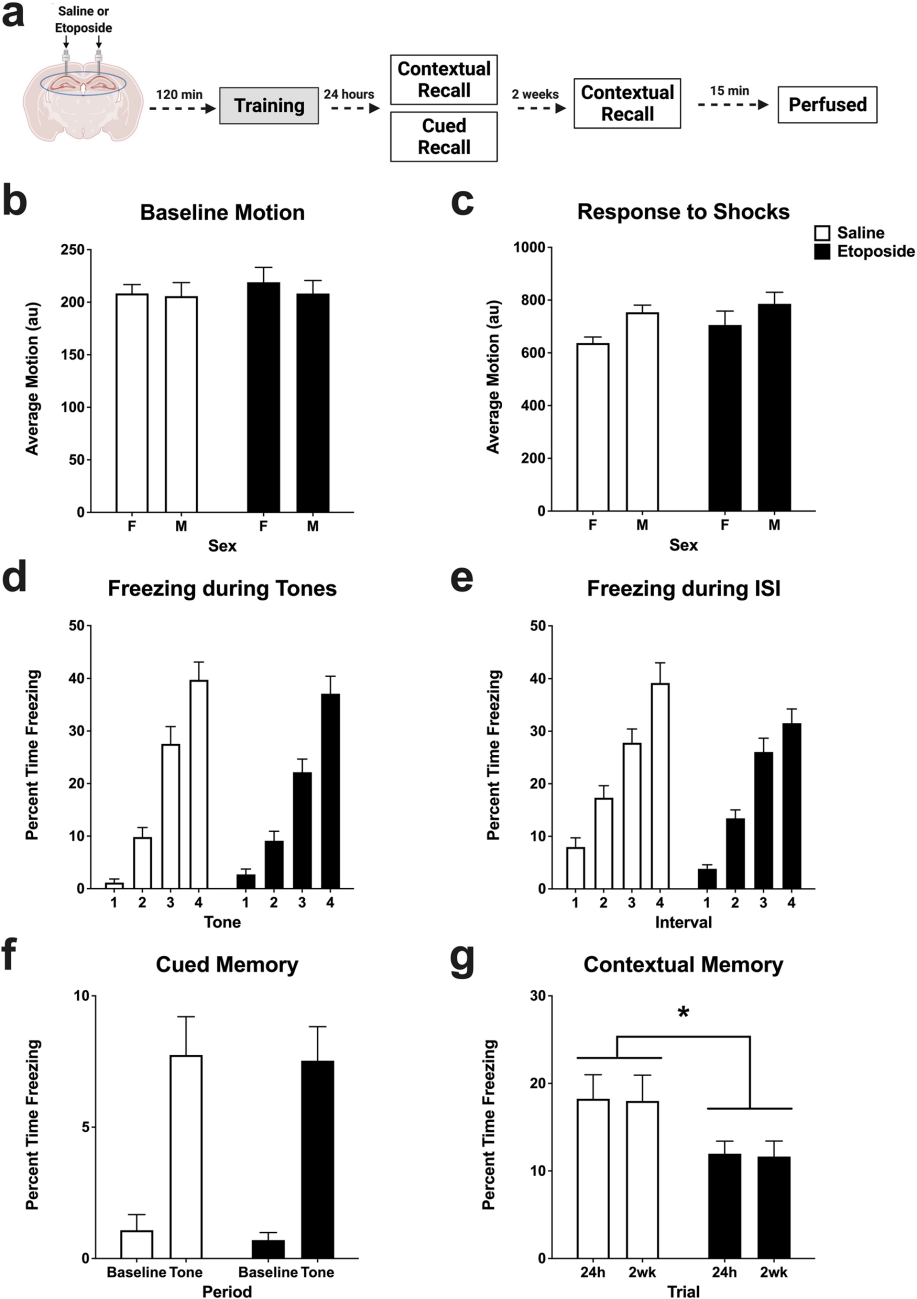
Behavioral performance in fear conditioning, cued, and contextual tests. (**a**) Schematic of the experimental design (Training: *n* = 10–24/sex/treatment; Cued & Contextual Recall: *n* = 10–13/sex/treatment). (**b**) Average motion (au) during the 2-min baseline period in fear training. No differences were detected based on treatment (*p* = 0.653) or sex (*p* = 0.649). (**c**) Average motion during the shocks (au). Males moved more during the shocks than females (*F*(1,66) = 5.125, *p* = 0.027), but no differences were detected based on treatment (*p* = 252). (**d**) Percent time freezing during the 4 tones. All animals increased over the course of training (*F*(2.382,161.992) = 131.083, *p* < 0.001). with no differences found between treatment (*p* = 0.455) or sex (*p* = 0.816). (**e**) Percent time freezing during the 4 inter-stimulus intervals. All animals increased over the course of training (*F*(2.260,153.674) = 113.594, *p* < 0.001), with no differences found between treatment (*p* = 0.108) or sex (*p* = 0.708). (**f**) Percent time freezing at baseline and during the tone in the 24 h cued recall test. All animals showed increased freezing in response to the tone (*F*(1,43) = 54.312, *p* < 0.001); no differences were detected based on treatment (*p* = 0.823) or sex (*p* = 0.771). (**g**) Percent time freezing during the contextual recall tests at 24 h and 2 weeks post-training. Mice that received etoposide infusions froze less at both time points than saline-infused mice (*F*(1,43) = 5.112, *p* = 0.029). No differences were seen based on sex (*p* = 0.522). All data are presented as mean ± SEM. *p < 0.05.

To assess if there was a relationship between cFos and Arc, we correlated signals in each region. There was no relationship between the number of cFos + and Arc + cells in the CA1 or CA3 regions. However, there was a positive correlation between the number of immunopositive cFos and Arc cells in the DG in saline-infused mice only (*p* < 0.001; Fig. 2l); there was no correlation in etoposide-infused mice (*p* = 0.772). We also correlated the number of γH2Ax foci with the number of cFos + cells in each region. There were no significant correlations in the CA3 or DG, though we found significant positive correlations in the CA1 of both saline- (*p* = 0.0176) and etoposide-infused (*p* = 0.0239) mice that underwent fear conditioning (Fig. 2m). There was no significant correlation in the CA1 of behaviorally naïve animals.

Altogether, these data suggest that hippocampal infusion of etoposide blunts cFos expression, and blunts Arc in the DG but increases it in the CA3 region.

### Hippocampal injection of etoposide impairs long-term contextual, but not cued, fear memory in both males and females

Based on the observed molecular changes in the hippocampus, we subsequently assessed long-term hippocampus-dependent and -independent memory. Our previous work indicated that systemic injections of etoposide affected males and females differently^13^, thus we assessed long-term memory in both males and females here (*n* = 13–15/sex/treatment). All animals went through the same infusion and fear training timeline and were subsequently tested for contextual (hippocampus-dependent) and cued (hippocampus-independent) memory at 24 h and contextual fear memory at 2 weeks (Fig. 3a). No differences were detected in body weight based on drug treatment (*p* = 0.828). Males weighed more than females throughout the study (*p* < 0.001; Table 1).

There were no differences in performance during fear training based on treatment. All animals showed similar activity levels at baseline (*p* = 0.653; Fig. 3b), motion in response to the shocks (*p* = 0.252; Fig. 3c), percent time freezing during the tones (*p* = 0.643; Fig. 3d), and percent time freezing during the inter-stimulus intervals (*p* = 0.127; Fig. 3e). Additionally, there were no differences between males and females except for the response to shock, where males moved more in response to the shock than females (*p* = 0.027; Fig. 3c).

Twenty-four hours later, we tested the mice for recall of cued memory, 3 h after the first contextual recall test (described below). Analysis of the response to the tone during the cued recall test indicated that all animals recognized the tone, shown by the increase in percent time freezing in all groups when the tone was played (*p* < 0.001; Fig. 3f). There were no effects of treatment (*p* = 0.823), sex (*p* = 0.905), or interaction between treatment and sex (*p* = 0.771), suggesting that non-hippocampus-dependent learning and memory was unaffected by our etoposide infusions into the CA1 region.

We also tested the animals for recall of contextual memory at 24 h, and again 2 weeks after training to include assessments of long-term effects of etoposide on hippocampus-dependent memory. There was an effect of treatment, with the animals that received etoposide freezing less at both time points compared to those that received saline (*p* = 0.029; Fig. 3g). There were no differences between the sexes (*p* = 0.522), and no interaction between treatment and sex (*p* = 0.659).

Lastly, we euthanized these animals 15 min after the last contextual test to analyze γH2Ax foci in the CA1 region. There were no long-term differences in this DSB signal based on treatment (*p* = 0.534) or sex (*p* = 0.347; Table 2).

**Table 2:** γH2Ax foci normalized to DAPI area (μm) in the CA1 region of animals that were euthanized 15 min after the last contextual recall test.

Sex	Treatment	
	Saline	Etoposide
Male	0.34 ± 0.18	0.33 ± 0.18
Female	0.77 ± 0.29	0.52 ± 0.19

Data presented as averages ± SEMs.

Altogether, these data indicate that CA1 infusions of etoposide selectively impair long-term, hippocampus-dependent memory.

## Discussion

The results of this study provide evidence that DSBs are involved in regulating IEG expression and long-term memory. Etoposide infusions in the hippocampus impaired long-term contextual memory, which is hippocampus-dependent, but not cued memory, which is hippocampus-independent. Mice infused with etoposide did not have higher levels of cFos + cells 2 h after infusion compared to 7 h after infusion. All mice that underwent fear conditioning had more γH2Ax foci in the CA1, CA3, and DG than behaviorally naïve mice, though we observed interactions between behavioral condition and treatment in the CA3 and DG. Additionally, the number of Arc + cells was decreased in the DG and increased in the CA3 region following etoposide infusions.

DSBs play an important role in transcriptional elongation during cell division^19,20^ and generating molecular diversity in the immune system^21^, but in non-dividing cells (such as neurons) DSBs have been thought of as the result of damaging factors and signals for apoptosis^22^. However, our data add to a growing literature that suggest an adaptive function of DSBs in non-dividing cells via regulating gene expression related to learning and memory. Previous studies have shown that stimulation of electrical activity in cultured hippocampal neurons led to DSBs forming on promoter regions of a small subset of genes, which led to their subsequent up-regulation^9^. These genes—including *fos*, *Npas4*, and *Egr1*—are known to regulate synaptic plasticity and related protein synthesis pathways. In mice, increased levels of γH2Ax foci were observed in relevant brain regions following introduction to a novel environment^8^. Importantly, the increase in this DSB marker was transient: levels returned to baseline after 24 h. This same study also showed that mice carrying dominant Alzheimer’s disease genetic mutations had higher baseline levels of γH2Ax foci and those levels failed to return to baseline levels 24 h after introduction to the novel environment. These initial studies indicated that the precise timing and location of physiological DSBs may have a role in learning and memory and could be dysregulated in neurodegenerative disease.

Subsequent studies explored the role of DSBs across cell types in the mouse brain and in different fear conditioning paradigms. Mice tested in contextual fear conditioning showed increased DSBs in the hippocampus and medial prefrontal cortex, many of which occurred on or near genes important for regulating synaptic plasticity^10^. Both neuronal and non-neuronal cells displayed an increase in DSBs in that study; we did not assess cellular sub-types in this current study. Adding to the role of DSBs in fear learning and memory, direct infusion of 0.1 µg/µL etoposide into the mouse prelimbic area impaired trace fear conditioning and disrupted IEG expression^18^. However, 1 ng/µL of etoposide did not alter behavioral performance. Our results extend these findings, demonstrating that an intermediate dose is sufficient to alter DSBs and IEGs in the hippocampus. While our infusions were directly into the CA1 region, we also observed alterations in the CA3 and DG. One possibility is that etoposide itself spread to these regions and directly affected DSB formation and IEG expression. Another is that the intra-hippocampal signaling was altered due to changes in the CA1, thereby indirectly altering DSBs and IEGs in the CA3 and DG^23^. Intriguingly, we discovered an interaction between behavioral condition (naïve or fear conditioned) and treatment (saline or etoposide), where etoposide infusions decreased γH2Ax foci in the DG and CA3 in fear conditioned mice but increased foci in naïve mice. This relationship likely reflects the sensitivity of interfering with the proper timing for adaptive DSBs during periods of learning.

Notably, our results indicate that inducing DSBs in the CA1 region led to blunted IEG expression immediately following fear conditioning. Previous research has shown that etoposide blunted the second wave of IEG mRNA levels (5 h post-training) in the prelimbic area^24^. Ionizing radiation—which induces single and double-strand breaks—has been shown to both up- and down-regulate IEG expression depending on dose, tissue analyzed, and timing^25–27^. For example, 2 Gy of X-ray radiation in hippocampal cultured neurons decreased cFos and Egr1 mRNA compared to sham controls at 60 min, but 2 Gy + NMDA activation led to increased Egr1 mRNA^28^. The 2 Gy alone and the 2 Gy + NDMA activation also resulted in increased γH2Ax to similar levels. These data indicate that while DSBs may contribute to IEG expression, alterations to precise regulation and timing can both increase and decrease these genes. Our data here suggest that this dose of etoposide combined with fear conditioning led to a down-regulation, essentially preventing a “peak” like the one observed in the saline-infused, fear conditioned group. This lack of an increase is likely the driving factor in the statistical interactions. However, it is possible that other doses or timing could result in up-regulation, warranting further studies to explore this.

Moreover, our results increase understanding of the central effects of etoposide, which is commonly used in cancer treatment regimens^12,17,29,30^. Very little data exist on the possible side effects of etoposide, as cancer and treatment status confound direct ties to etoposide alone. Etoposide does cross the blood brain barrier, though not well, with only ~ 3% entering the brain^16^. Even in small amounts, the hippocampus may be particularly susceptible to etoposide, as etoposide reduced hippocampal polyamines^30^. However, hippocampus-dependent function was not assessed in that study. Research into combined cancer treatment suggest that the hippocampus is sensitive to chemotherapy: transient treatment with cyclophosphamide and doxorubicin in ovariectomized female rats impaired contextual, but not cued, fear memory^31^. Additionally, following chemotherapy in patients, hippocampal volume and connectivity are altered and correlate with impaired cognition^32,33^. Direct delivery of 0.5 mg of etoposide in the ventricles of patients (with peak CSF levels reaching 9 µg/mL) elicited side effects of headaches and meningitis; learning and memory were not assessed, though^17^. Our previous experiment showing that systemic injections of a medium dose of etoposide impaired contextual and cued fear memory tied etoposide specifically to learning and memory impairments^13^.

While IEGs generally tend to increase upon stimulation, there is task- and region-dependent specificity^34^. Our Arc and cFos cell count data were correlated only in the DG, but not in the CA1 or CA3 regions of the hippocampus. We also observed an increase in Arc in the CA3, in contrast to Arc in the DG and cFos in all sub-regions analyzed. cFos immunoreactivity was correlated in the hippocampal sub-regions analyzed, but Arc was not. Others have shown similar results, with region-, task- and time-specific increases in different IEGs^7,35,36^. Within the hippocampus, the CA1 is shown to be required for both acquisition and recall of contextual memory, while the CA3 is only required for acquisition^37^, and timing of context related IEG expression in the CA1, CA3, and DG are distinct^36^. Notably, *Arc* and *cFos* fall into two subcategories of IEGs: *Arc* is classified as an “effector” IEG, while *cFos* is classified as regulatory transcription factor (RTF)^34,38,39^. Thus, while related, others have found that Arc and cFos RNA levels in rats are correlated in some regions (such as the entorhinal cortex) but not others (like the hippocampus) following spatial learning^34^. This sub-categorization may account for the distinct effects we observed in the hippocampus. Even with this subtle difference, our results suggest that etoposide effects both sub-types of IEGs. Further research should continue to identify specific IEG families that are regulated by DSB-induced gene expression, as well as explore more brain regions and task-dependency.

In summary, these experiments indicate that induction of DSBs in the hippocampus interferes with learning and memory and that proper regulation of DSB timing and location is related to intact learning and memory. Future efforts are warranted to identify the role of DSBs in other forms of learning and other brain regions, such as the amygdala. Additionally, characterizing the effects of a wider range of doses will be important, especially when considering the translational relevance.

## Methods

### Animals

A total of 84 male (*n* = 57) and female (n = 27) C57Bl/6 J mice from Jackson Labs (Bar Harbor, ME) were delivered to OHSU at 5 weeks of age. Surgical cannula implantation occurred at 8–11 weeks of age. Infusion and behavioral testing occurred at 10–13 weeks of age. A total of 9 animals were euthanized due to surgical complications.

All animals were group housed at 5 mice/cage until the day of surgery. Animals were singly housed for 1-week after surgery to allow for recovery; after 1 week, animals were group-housed 3 mice/cage for the remaining duration of experiments. Body weights were recorded weekly starting the day of surgery.

All animal procedures were reviewed and approved by the Oregon Health & Science University IACUC and in accordance with AAALAC and ARRIVE guidelines. Researchers were blinded to the treatment groups throughout all experiments.

## Cannula surgery

Surgeries were performed according to our standard protocol^40^. Briefly, mice were treated with oral meloxicam (5 mg/kg) prior to being induced with 5% isoflurane and maintained at 1.5–2% isoflurane throughout surgeries. A water heating pad was used to maintain body temperature; depth of anesthesia and breathing were monitored. Mice were secured in a stereotaxic frame (World Precision Instruments, Sarasota, FL) with fixation bars. Ophthalmic ointment was applied to the eyes, and their heads were shaved. A local subcutaneous injection of lidocaine was given, and heads sterilized by alternating 3 swabs of betadine and 2 swabs of 70% isopropanol. An incision was made on the midline of the scalp to expose the skull and holes drilled bilaterally above the hippocampus (Bregma coordinates: AP: − 1.7 mm, ML: ± 1.0). A bilateral guide cannula (DV projection: 1.2 mm) was then lowered and secured in place with dental cement (Lang Dental Mfg. Co., Wheeling, IL). Once secured, a dummy cannula was inserted and mice removed from the stereotaxic frame. Mice received two days of post-op treatment with oral meloxicam (5 mg/kg).

## Infusions and fear conditioning

Etoposide phosphate (etopophos) powder was purchased through the OHSU research pharmacy (E.R. Squibb & Sons, LLC, New Brunswick, NJ). It was diluted in saline to a 20 mg/mL stock solution and stored at − 80 °C until the day before use. The working solution was diluted to a final concentration of 0.1 µg/µL. This dose was chosen based on the previous use of direct infusions of etoposide in the prelimbic area of mice^18^, as well as clinical data for common doses used in patients^17^ and accounting for toxicity levels^15^.

For infusions, mice were lightly anesthetized with 5% isoflurane. Mice were removed from anesthesia and gently held by the researcher. Internal cannula were then inserted; 0.3 µL of saline or etoposide were infused in each hemisphere at a rate of 0.3 µL/min. Internal cannula were left in situ for 60 s before mice were placed back into the home cage.

Fear conditioning was conducted 2 h after infusions. We implemented a common protocol previously used by our lab^13^. Briefly, mice were placed in to fear conditioning chambers (MedAssociates, Fairfax, VT) for a baseline period of two minutes before being exposed to 4 tones that co-terminated with a 2 s, 0.5 mA shock. There were 90 s inter-stimulus intervals between each tone-shock pairing. Upon completion, mice were returned to their home cages and fear chambers cleaned with 0.5% acetic acid.

For assessment of long-term hippocampus-dependent and -independent memory, mice were tested in contextual and cued fear conditioning 24 h after training. The contextual trial (hippocampus-dependent) was a 5-min period in the same chambers as training; no tones were presented, and chambers were cleaned with 0.5% acetic acid. For the cued trial (hippocampus-independent), contextual clues were eliminated by inserting a new floor, walls, and roof, as well as cleaning with 10% isopropanol. The test was comprised of a 90 s baseline period followed by a 3-min presentation of the same tone played during training. There was a 3 h period between the contextual and cued recall tests. Lastly, we assessed contextual memory again at 2 weeks to explore the lasting effects of hippocampal etoposide infusions^13^.

## Tissue collection

We randomly split our animals into two experimental categories: the “immediate” cohorts and the “long-term” cohorts.

To assess effects of infusions on γH2Ax foci and IEGs, a cohort of male mice received infusions, were trained in fear conditioning, and euthanized immediately post-training (2 h after infusion, *n* = 4/treatment) or 5 h post-training (7 h after infusion, *n* = 7–8/treatment; Fig. 1a, 2a). These times were chosen as peak IEG signal ranges from 15 to 120 min after stimulation and typically return to baseline levels around 4 h later^41–43^. Additionally, direct infusions of etoposide were shown to lead to increased IEG expression 5 h after infusion^18^. For analysis of long-term effects of infusions on γH2Ax, separate cohorts of male and female mice were euthanized 15 min after completion of the 2-week contextual recall test (*n* = 13–15/sex/treatment; Fig. 3a).

All animals were euthanized by perfusion. Mice received an *i.p.* injection of a ketamine-xyline cocktail. Once animals were non-responsive, they were perfused with ice-cold 1X PBS followed by 4% paraformaldehyde (PFA). Brains were removed and soaked in 4% PFA overnight, then sunk in 30% sucrose solution. Brains were then sectioned at 40 μm thickness using a cryostat; the lower left hemisphere was notched to indicate side when sectioning. Sections were stored in cryopreserve until use.

## Immunohistochemistry

### cFos and Arc immunohistochemistry

Free floating sections (*n* = 4/animal) were stained for cFos and Arc using a standard 3,3′-diaminobenzidine tetrahydrochloride (DAB) protocol^44,45^. Briefly, tissue was rinsed 3 × 5 min in 1 × PBS and activated with 0.3% hydrogen peroxide for 15 min. Tissue was again rinsed 3 × 5 min in 1 × PBS and blocked in 4% normal goat serum (NGS) in 0.3% PBS-Triton X. Sections were then incubated in primary antibody diluted in 4% NGS for approximately 48 h at 4 °C (rabbit anti-cFos: 1:5000, Santa Cruz, sc-52, lot #G2612; mouse anti-Arc: 1:300, Santa Cruz, sc-17839, lot #K1609). Tissue was washed 3 × 5 min before incubating in secondary antibody diluted in 4% NGS for 1 h (goat anti-rabbit biotinylated: 1:200, Vector Labs, BA-100, lot #ZH0615; goat anti-mouse biotinylated: 1:200, Vector Labs, BA-9200, lot #X0623). After 3 × 5 min washes, avidin–biotin complex was applied for 1 h (Vector labs), and washed 3 × 5 min again. Chromagen was applied for exactly 10 min and the reaction stopped with deionized water, washed 2 × 5 min in 1 × PBS, and slide mounted. Slides were coverslipped with Cytoseal-60 after going through an ethanol-xylene dehydration series.

### γH2Ax Immunofluorescence

Free floating sections (*n* = 4 sections/animal) were stained for γH2Ax foci using our standard 3-day fluorescence protocol^46^. Briefly, tissue was rinsed 3 × 5 min in 1 × PBS and blocked for 1 h in 4% NGS. Sections were incubated overnight at room temperature in rabbit-anti-γH2Ax (1:1000, Cell Signaling, 97,185, lot #17), rinsed 3 × 5 min in 1 × PBS, then incubated overnight in goat-anti-rabbit Alexa 594 secondary (1:1000, Invitrogen, A11012, lot #1,892,265). Tissue was again rinsed 3 × 5 min in 1 × PBS, then incubated for 20 min in a DAPI counterstain (1:200, Sigma Aldrich, St. Louis, MO). After another 20 min rinse in 1 × PBS, tissue was mounted and coverslipped with Citifluor CFMR2 antifade mounting media (Electron Microscopy Sciences, Hatfield, PA, #17,979–10).

### Fluoro-Jade C

To confirm that infusions did not cause widespread cell death, we stained sections for Fluoro-Jade C as previously described^47,48^. Four hippocampal sections per animal were slide mounted and dried for 2 h on a slide warmer at 55 °C. Slides were immersed for 5 min in 80% EtOH with 1% NaOH, then 2 min in 70% EtOH with 1% NaOH. Following a 2 min rinse in ddH_2_O, slides were incubated for 15 min in 0.06% KMnO_4_, then rinsed again for 2 min in ddH_2_O, and incubated for 15 min in 0.0001% Fluoro-Jade C (16A, Histo-Chem, Inc., AR) dissolved in 0.1% acetic acid. Slides were rinsed 3 × 1 min in ddH_2_O, and allowed to air dry overnight in the dark. Slides were cleaned in xylene, then cover slipped with Permount™ mounting medium (Fisher Scientific, MA).

## Microscopy and quantification

For all DAB-stained sections, images were captured with an upright Olympus IX81 microscope (Olympus, Center Valley, PA, USA) equipped with CellSens imaging software. Images of each sub-region of the hippocampus were taken at 10 × magnification with 100 ms exposure time. cFos- and Arc-positive cells were identified by the dark nuclear label and counted using ImageJ software (NIH, Bethesda, MD). Fluoro-Jade C sections were also captured using the Olympus IX81 upright microscope at 10 × magnification with 5 s exposure time using a FITC filter.

For γH2Ax-stained fluorescent sections, images were captured with a Zeiss LSM 980 FastAiry microscope (Zeiss, Thornwood, NY, USA) equipped with Zen imaging software. Images of each sub-region of the hippocampus were taken at 63 × magnification in oil immersion. Analysis of γH2Ax foci was performed using ImageJ software. The number of foci was normalized to the area occupied by DAPI for analysis^46^.

## Statistics and reproducibility

All data were assessed for normality prior to proceeding with parametric tests. Statistical analyses were conducted using Prism v. 7 (GraphPad, San Diego, CA) and SPSS v 27 (IBM, Armonk, NY) software.

For fear conditioning, the average motion (au) during the initial baseline period was analyzed with a 2-way ANOVA. The average motion (au) during the shocks, percent time freezing during the tones, and percent time freezing during the inter-stimulus intervals were analyzed using a repeated measures ANOVA with sex and treatment as between-group variables and time as the within-group variable.

For all immunohistochemical analyses, hemisphere was initially included as a variable to confirm there were no hemisphere-dependent differences. In all cases, there were no significant effects or interactions with hemisphere, so it was dropped from the statistical model.

For γH2Ax foci analysis, the number of γH2Ax foci normalized to the DAPI area (μm) was averaged in each section. We then used a repeated measures ANOVA with treatment as the between group variable and section as the within-group variable for analysis.

For analysis of the number of *Arc* and *cFos* positive cells, we analyzed the average number of immunopositive cells per section using a repeated measures ANOVA with treatment and time post-training as between-group variables and section as the within-group variables. Sidak’s post hoc test was used following these ANOVAs.

## Supplementary Information

Supplementary Information 1.

Supplementary Information 2.

Supplementary Information 3.

Supplementary Information 4.

## Data Availability

The datasets generated during and analyzed during the current study are available from the corresponding author on reasonable request.
