# Supplementary figures and images for: Infusion of etoposide in the CA1 disrupts hippocampal immediate early gene expression and hippocampus-dependent learning
Source: Sci Rep. 2022 Jul 27;12:12834. doi: 10.1038/s41598-022-17052-y (PMC9329441; doi:10.1038/s41598-022-17052-y)

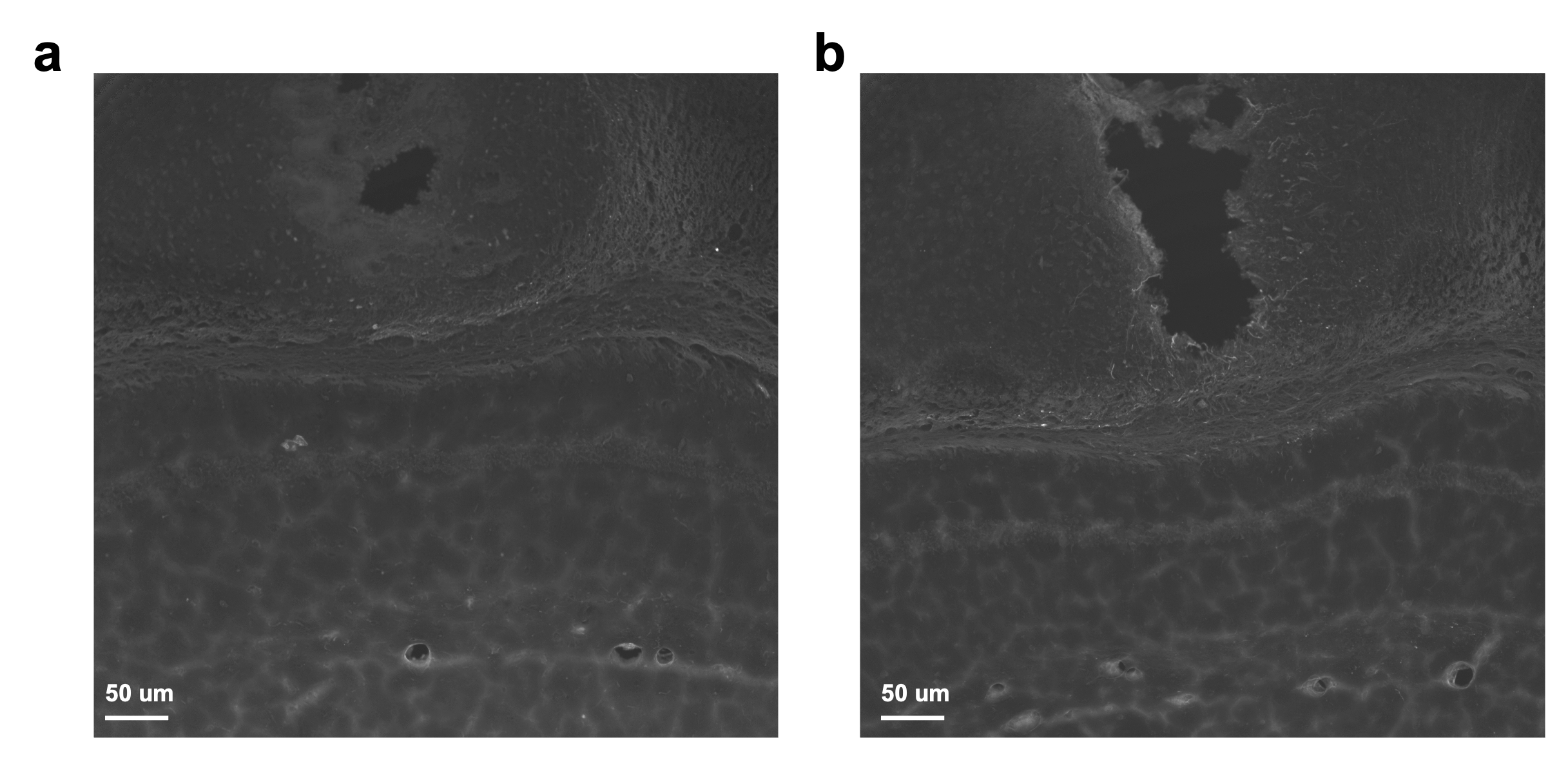

Supplement: Supplementary file 1 — Supplementary Information 1. [file 41598_2022_17052_MOESM1_ESM.png]

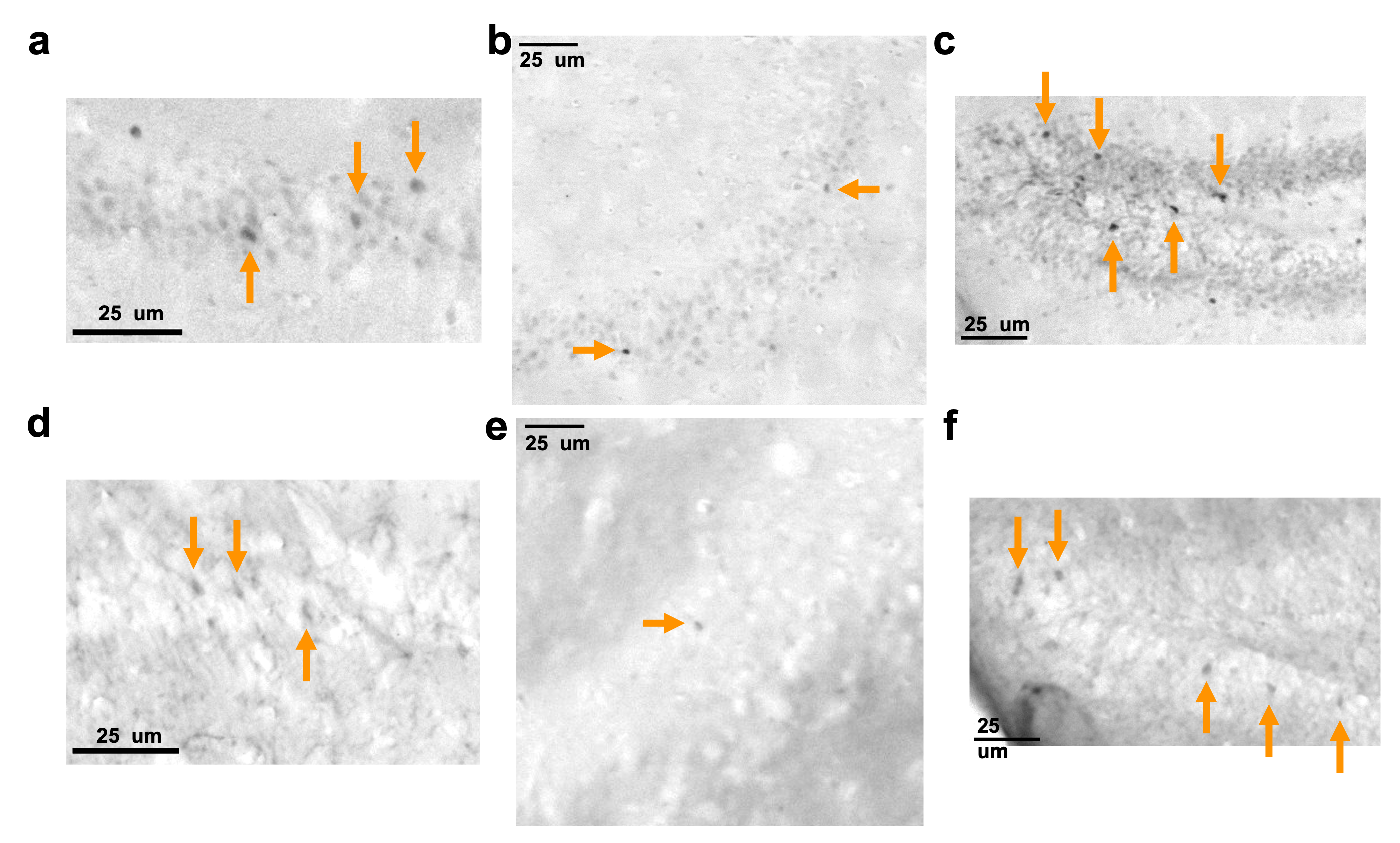

Supplement: Supplementary file 2 — Supplementary Information 2. [file 41598_2022_17052_MOESM2_ESM.png]

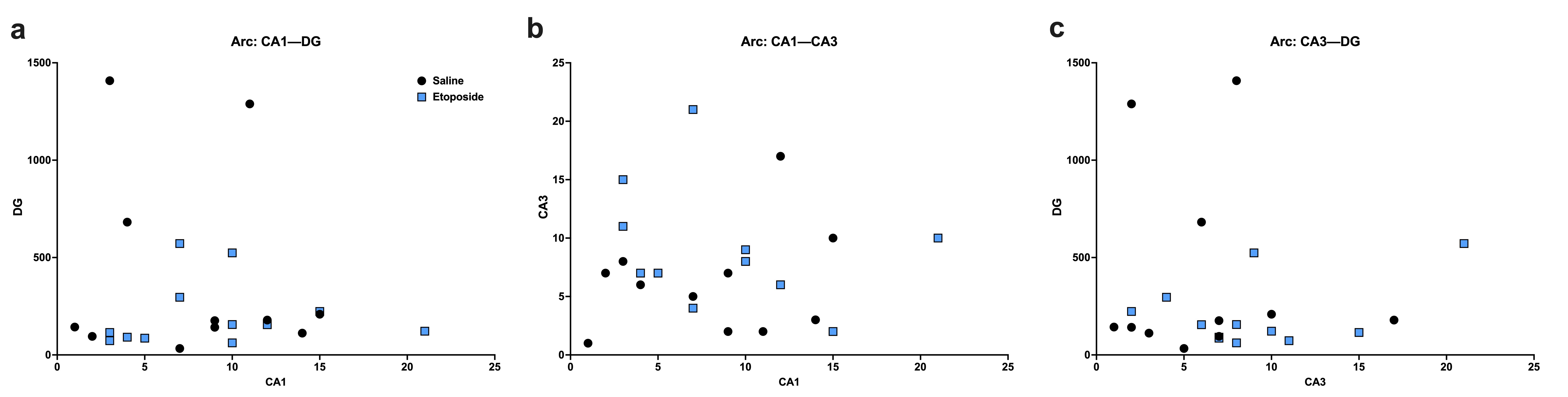

Supplement: Supplementary file 3 — Supplementary Information 3. [file 41598_2022_17052_MOESM3_ESM.jpg]
