# Supplementary material for: Infusion of etoposide in the CA1 disrupts hippocampal immediate early gene expression and hippocampus-dependent learning
Source: Sci Rep. 2022 Jul 27;12:12834. doi: 10.1038/s41598-022-17052-y (PMC9329441; doi:10.1038/s41598-022-17052-y)
Supplement: Supplementary file 4 — Supplementary Information 4. [file 41598_2022_17052_MOESM4_ESM.docx]

**Supplemental Figure 1.** No Fluoro-Jade C staining in the CA1 region of the hippocampus in **A)** a saline-infused animals and **B)** an etoposide-infused animal. There was no observable indication of cell death in any animals.

**Supplemental Figure 2**. Representative images of cFos staining in **A)** the CA1, **B)** the CA3, and **C)** the DG. Representative images of Arc staining in **D)** the CA1, **E)** the CA3, and **F)** the DG. Orange arrows point to examples of cells that were counted as immuno-positive.

**Supplemental Figure 3**. No correlation of total number of Arc+ cells between the **A)** CA1 and DG, **B)** CA1 and CA3, and **C)** CA3 and DG.
